# Reproductive resource partitioning in two sympatric *Goniothalamus* species (Annonaceae) from Borneo: floral biology, pollinator trapping and plant breeding system

**DOI:** 10.1038/srep35674

**Published:** 2016-10-21

**Authors:** Jenny Y. Y. Lau, Chun-Chiu Pang, Lawrence Ramsden, Richard M. K. Saunders

**Affiliations:** 1 School of Biological Sciences, The University of Hong Kong, Pokfulam Road, Hong Kong, China

## Abstract

The floral phenology, pollination ecology and breeding systems of two sympatric early-divergent angiosperms, *Goniothalamus tapisoides* and *G. suaveolens* (Annonaceae) are compared. The flowers are protogynous and morphologically similar, with anthesis over 23–25 h. Both species are predominantly xenogamous and pollinated by small beetles: *G. tapisoides* mainly by Curculionidae and *G. suaveolens* mainly by Nitidulidae. Coevolution and reproductive resource partitioning, reducing interspecific pollen transfer, is achieved by temporal isolation, due to contrasting floral phenologies; and ethological isolation, due to contrasting floral scents that contain attractants specific to the two beetle families. Analysis of floral scents revealed three volatiles (3-methylbutyl acetate, ethyl hexanoate and 2-phenylethanol) that are known to be nitidulid attractants in the floral scent of *G. suaveolens*, but absent from that of *G. tapisoides*. An effective pollinator trapping mechanism is demonstrated for both species, representing the first such report for the family. Trapping is achieved by the compression of the outer petals against the apertures between the inner petals. This trapping mechanism is likely to be a key evolutionary innovation for *Goniothalamus*, increasing pollination efficiency by increasing pollen loading on beetles during the staminate phase, promoting effective interfloral pollinator movements, and increasing seed-set by enabling rapid turn-over of flowers.

Reproductive interference can be defined as the fitness cost resulting from sexual interactions between different species^1^,^2^,^3^. This is commonly manifested in plants as interspecific pollen transfer between sympatric species, and can lower reproductive efficiency due to competition for pollinators, pollen wastage, clogging of the stigma surface with alien pollen (hence impeding successful germination of compatible pollen), and the formation of sterile or poorly adapted hybrids^4^,^5^,^6^. Although many studies have focused on the deleterious consequences arising from reproductive interference^7^,^8^,^9^, other research has suggested that the increased flowering density observed in such species can be beneficial as it increases pollinator abundance^10^.

Various reproductive isolation mechanisms, including mechanical and ethological isolation, are known to reduce interspecific pollen transfer in sympatric species^10^,^11^. Mechanical isolation is generally achieved by structural adaptations to the flowers of competing species to ensure the deposition/retrieval of pollen from different parts of the body of the shared pollinator^2^,^6^,^12^, or through the evolution of species- or genus-specific floral morphologies that are adapted for different pollinator guilds^11^,^13^. Ethological isolation is commonly achieved following adaptations to floral shape, pigmentation and scent^11^: although this can be a direct consequence of mechanical isolation^11^, it can also be due to changes in floral scent chemistry that favour different pollinators^14^,^15^,^16^, and the ‘flower constancy’ behaviour of some pollinators in which the pollinator makes repeated visits to the same plant population^8^,^17^. Reproductive isolation is widely believed to be a major driver of floral evolution, enhancing pollination efficiency by reducing interspecific pollen transfer and hence maximising resource partitioning^2^,^10^,^18^.

Many studies of reproductive interference and the evolution of reproductive isolation between sympatric species have focused on taxa with structurally complex flowers that maintain a high degree of pollinator specificity. None of the published studies focus on early-divergent angiosperms, which are typically pollinated by representatives of a single pollinator guild rather than showing species-level specificity. The pantropical flowering family Annonaceae— a major component of tropical lowland forests^19^, comprising c. 108 genera and 2,400 species of trees and woody lianas^20^—is an ideal focus for such studies. Most Annonaceae species are pollinated by small beetles (Curculionidae, Nitidulidae and Staphylinidae, and smaller Chrysomelidae), although other pollinator guilds have been identified in some derived lineages, including large beetles (Scarabaeidae subfam. Melolonthinae and Dynastinae), thrips, flies, bees and cockroaches^21^. Although most Annonaceae flowers are visited by species belonging to a single pollinator guild, many studies report visits by multiple different representatives of that guild^21^. It is furthermore common for a single pollinating species to visit more than one species of Annonaceae: the nitidulid species *Carpophilus plagiatipennis* (Motschulsky), for example, has been reported to pollinate flowers of both *Monoon coffeoides* (Thwaites ex Hook. f. & Thomson) B. Xue & R.M.K. Saunders (as ‘*Polyalthia coffeoides*’)^22^ and *Goniothalamus gardneri* Hook. f. & Thomson^21^.

The present study focuses on *Goniothalamus*, one of the largest tree genera in Annonaceae, with c. 134 species^23^. *Goniothalamus* flowers are pendent and hermaphroditic, with a tripartite perianth comprising a trimerous calyx and two whorls of three petals. The inner petals are apically connivent and postgenitally fused over the reproductive organs, with three apertures located between the basal ‘claws’ of contiguous petals, close to the floral receptacle; these apertures are generally blocked by the alternately positioned outer petals, although subsequent movement of the outer petals exposes the apertures, allowing pollinators access. Existing knowledge of the reproductive ecology of *Goniothalamus* is very limited, although there are published reports on: *G. australis* Jessup^24^; *G. gardneri*^21^; *G. wightii* Hook. f. & Thomson^25^; and ‘*G. uvarioides*,’ *G. velutinus* Airy Shaw and an unidentified species^26^,^27^,^28^. These studies have revealed that the flowers are protogynous with anthesis extending over two days, and are pollinated by curculionid and nitidulid beetles that are likely to be attracted to the flowers by the fruity scent.

*Goniothalamus* species are often conspicuous components of tropical lowland forests and it is likely that congeners co-occur in close proximity. Two Bornean endemic species, *G. tapisoides* Mat-Salleh and *G. suaveolens* Becc. are widely sympatric, possessing largely overlapping distributional ranges (Fig. 1) and occupying similar habitats (dipterocarp forests at elevations up to 1140 m and 850 m, respectively^29^,^30^). Our fieldwork in Brunei has revealed that these two species commonly co-exist immediately adjacent to each other and have overlapping peak flowering seasons: although both species flower throughout the year, herbarium records indicate that flowering is particularly intense in *G. tapisoides* from May until August, and in *G. suaveolens* from May until September.

Our study aims to describe the floral phenology and pollination ecology of the genus in detail for the first time, based on field observations of *G. tapisoides* and *G. suaveolens* in Brunei. This data not only provides a valuable contribution to our knowledge of the diversity and evolution of pollination systems in this species-rich Annonaceae lineage, but also enables an investigation of possible reproductive resource partitioning in response to reproductive interference. This is the first empirical study that evaluates data on floral phenology, pollinator composition, floral scent and breeding system in two closely related sympatric Annonaceae species.

## Materials and Methods

### Study species

**Figure 2:**
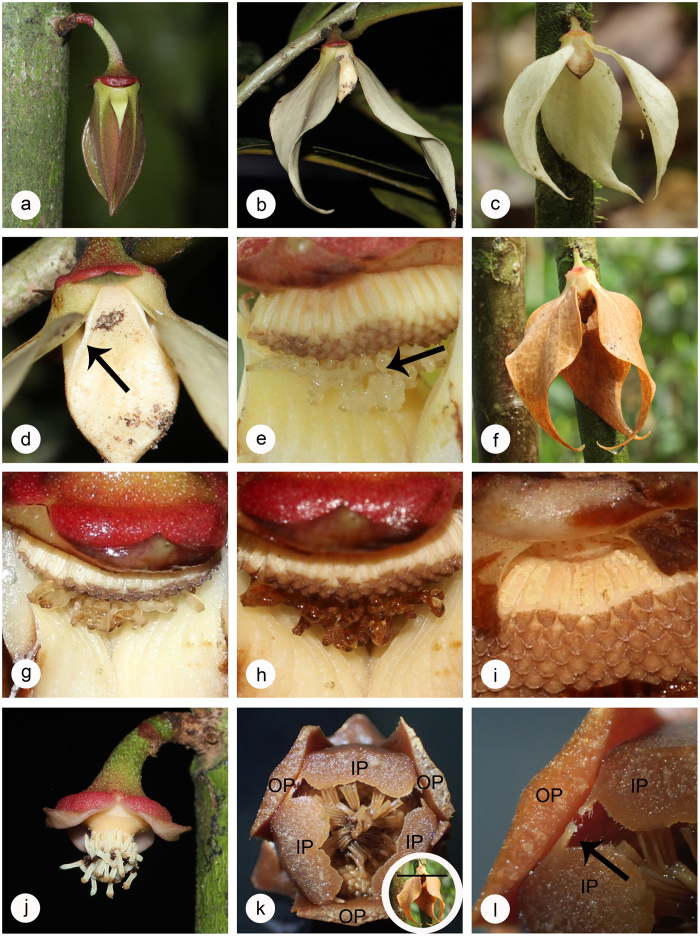
Photos of flowers of *Goniothalamus tapisoides* during different phenological stages. (**a)** early bud stage; (**b**) rising of outer petals; (**c**) pistillate phase flower (outer petals rise); (**d**) opening of basal apertures during pistillate phase; (**e**) secretion of stigmatic exudate during pistillate phase; (**f**) closing of basal apertures at the end of pistillate phase; (**g**) drying of stigmatic exudate during interim phase; (**h**) changing colour of stigmatic heads during interim phase; **(i**) staminate phase; (**j**) end of staminate phase; **(k**) transverse section during staminate phase showing trapping mechanism: OP = outer petal, IP = inner petal, black line showing where the transverse section was cut; (**l**), flanges of the inner petals.

**Figure 3:**
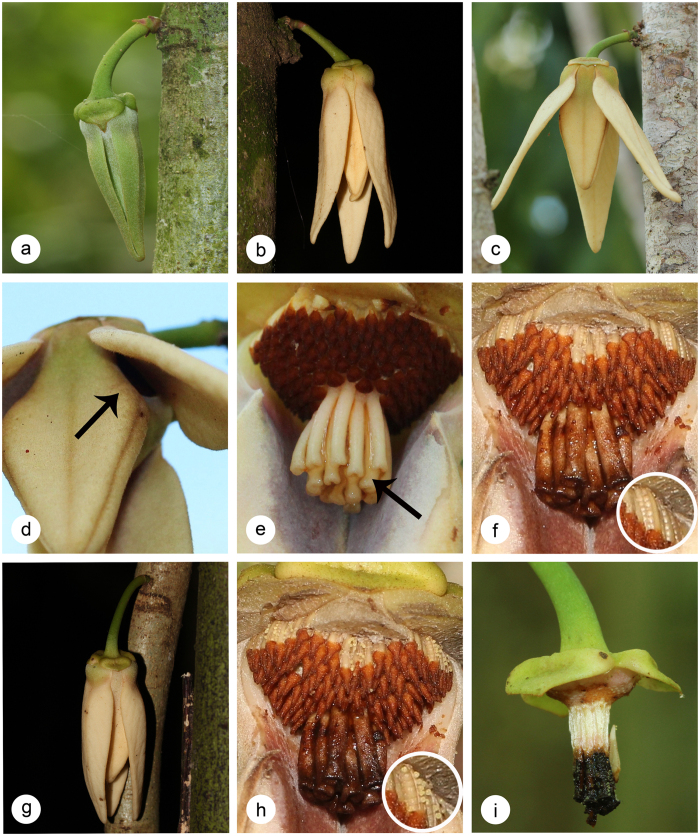
Photos of flowers of *Goniothalamus suaveolens* during different phenological stages. (**a**) early bud stage; (**b**) late bud stage; (**c**) rising of outer petals; (**d**) opening of basal apertures during pistillate phase; (**e**) secretion of stigmatic exudate during pistillate phase; (**f**) change of stigmatic exudate during interim phase; (**g**) closing of basal apertures; (**h**) staminate phase; (**i**) end of staminate phase.

*Goniothalamus tapisoides* (Fig. 2; inclusive of *G. longistipites* Mat-Salleh and *G. sinclairianus* Mat-Salleh^30^) and *G. suaveolens* (Fig. 3; inclusive of *G. cylindrostigma* Airy Shaw^30^) are small tree species, growing to c. 10 m^30^. The flowers of both species are typical for the genus, with apically connivent inner petals with basal apertures that are blocked by the outer petals. The petals are cream-coloured in both species, but differ in size and thickness^30^: the outer petals of *G. suaveolens* are smaller (30–45 × 6–11 mm) and more fleshy than those of *G. tapisoides* (40–60 × 15–30 mm); whereas the inner petals of *G. suaveolens* are considerably larger (22–26 × 8–9 mm) than those of *G. tapisoides* (9–11 × 3–6 mm). Both species have bisexual flowers with numerous stamens and unfused carpels.

### Study sites

The fieldwork was undertaken in Brunei Darussalam (permit 53/JPH/BOT/02 PT.3, issued in 2013 and renewed in 2014 by Forestry Department, Ministry of Industry and Primary Resources, Bandar Seri Begawan, Brunei Darussalam, with export permit BioRIC/HOB/TAD/51–22). Brunei has a hot tropical climate with average maximum and minimum annual temperatures of 32.1 °C and 23.8 °C, respectively, and with an average annual rainfall of 2770 mm^31^,^32^.

All field observations were conducted in well preserved localities, largely free from anthropogenic disturbance. *Goniothalamus tapisoides* was studied at four sites, viz.: (1) Jalan Rampayoh Timur, Labi, Belait District (04°22′03.6″N, 114°27′37.3″E; 59 m elevation); (2) Andulau Forest Reserve, Belait District (04°38′27.1″N, 114°30′33.3″E; 59 m elevation); (3) Bukit Teraja, Labi, Belait District (04°19′34.9″N, 114°25′52.3″E; 58 m elevation); and (4) Beelaban, Temburong District (04°40′19.4″N, 115°05′06.8″E; 47 m elevation). Three populations of *Goniothalamus suaveolens* were similarly selected, viz.: (1) Jalan Rampayoh Timur, Labi, Belait District (04°22′05.9″N, 114°27′38.4″E; 59 m elevation); (2) Beelaban, Temburong District (04°40′24.3″N, 115°05′02.9″E; 37 m elevation); and (3) Kuala Biang, Temburong District (04°40′06.0″N, 115°06′15.2″E; 68 m elevation). Two of these sites (Jalan Rampayoh Timur and Beelaban) therefore had sympatric populations of *G. suaveolens* and *G. tapisoides* (more than 100 individuals of each species observed in each site).

Voucher specimens of *G. tapisoides* were collected in Jalan Rampayoh Timur (*Y.Y. Lau LYY002*, 6 May 2013), Andulau Forest Reserve (*Y.Y. Lau LYY107*, 22 May 2014), Bukit Teraja (*Y.Y. Lau LYY003*, 8 May 2013) and Beelaban (*Y.Y. Lau LYY104*, 14 May 2014). Voucher specimens of *G. suaveolens* were collected in Jln Rampayoh Timur (*Y.Y. Lau LYY004*, 16 May 2013), Beelaban (*Y.Y. Lau LYY102*, 13 May 2014) and Kuala Biang (*Y.Y. Lau LYY103*, 14 May 2014). All specimens were deposited in Brunei Darussalam Herbarium.

### Floral phenology

Floral phenological observations were undertaken over two consecutive flowering seasons in 2013 and 2014. Forty flower buds from 16 individuals of *G. tapisoides* and 30 flower buds from 16 individuals of *G. suaveolens* were tagged and monitored during April and May in both years. Detailed timing of the onset and cessation of floral sexual stages were recorded over 10 days at 1-hr intervals beginning immediately before the onset of the pistillate phase and continuing until the end of the staminate phase. The following features were monitored throughout anthesis: changes in floral organ colour, presence of stigmatic exudate, movement of petals and associated opening and closing of the apertures in the floral chamber, and wilting and abscission of floral organs. Stigmatic receptivity were assessed using 3% hydrogen peroxide (H_2_O_2_) solution, in which the formation of gas bubbles is indicative of peroxidase enzyme activity^33^. Stigmatic receptivity was noted to be associated with visible changes in the presence of stigmatic exudate and stigmatic colour change, providing a logistically easier indicator of receptivity. The onset of the staminate phase was indicated by anther dehiscence.

Flower-level phenological data were assessed for multiple flowers in each individual tree, enabling the phenological pattern at the individual plant level (the number of pistillate-phase and staminate-phase flowers) to be determined and hence, by concatenating the data from ten tagged individuals of each species, enabling population-level phenological patterns to be deduced. This information enables an assessment of intra- and inter-individual floral synchrony.

### Pollinators and floral visitors

Surveys of floral visitors were conducted on 200 flowers of *G. tapisoides* and 30 flowers of *G. suaveolens* during April and May in 2013 and 2014. The activities of floral visitors were observed and recorded at 1-hr intervals. Representative samples were caught with an entomological net, immobilized with chloroform-soaked paper in Eppendorf tubes, and then dried using silica gel. The adherence of pollen grains on the insect’s body was assessed. Taxonomic identification was undertaken by entomologists at the Natural History Museum, London.

The assessment whether floral visitors were effective pollinators was based on the following criteria: relative visitation rate; coincidence of visits with the duration of the pistillate and staminate floral phases; attachment of pollen grains to the insect; and evidence of interfloral movement^34^. Since *Goniothalamus* species are known to be protogynous, the lattermost criterion was indicated by observing pollen-laden insects in pistillate-phase flowers.

### Floral thermogenesis and scent chemistry

Temperatures inside the pollination chamber of five flowers of each *Goniothalamus* species were measured using a digital temperature data logger (Testo 176-T4, Testo, New Jersey) with type-K thermocouples (±0.3 °C accuracy). Measurements were taken at 5-min intervals, beginning before the onset of the pistillate phase and continuing beyond the end of the staminate phase. Ambient temperatures immediately outside the pollination chamber were recorded simultaneously using the same data logger and thermocouples after cross-calibration.

Floral scents were sampled separately using immature flowers and pistillate-, interim- and staminate-phase anthetic flowers. The flowers were collected and sealed in previously unused polypropylene bags, and a solid-phase microextraction (SPME) fibre with a 65-μm divinylbenzene/polydimethylsiloxane coating inserted for at least 2 hours to adsorb volatile compounds. Air was also sampled from an empty polypropylene bag as a control, enabling any volatiles identified from the sample to be excluded from the floral scent analyses. Gas chromatography-mass spectrometry (GC-MS) was used to analyse the SPME fibres, using an Agilent 6890N gas chromatograph (Agilent Technologies, Palo Alto, California) coupled with an Agilent 5973 mass selective detector with a 30 × 0.255 mm i.d. DB-WAX capillary column and a 0.25 mm film (J & W Scientific, Folsom, California), with helium as the carrier gas. The SPME fibre was inserted into the inlet for 1 minute at 250 °C to allow vaporization of volatiles; the oven was then maintained at 50 °C for 5 min, and subsequently increased at 5 °C per minute until reaching 230 °C. Verification of chemicals was achieved by comparing retention times of selected pure compounds.

The NIST 2014 MS library bundle (National Institute of Standards and Technology, Gaithersburg, Maryland) was used to determine compound identity, although compounds with estimated identity likelihoods <80% were considered as unknown. Chemicals that were recorded in different floral phases (non-receptive, pistillate, interim and staminate) were listed after excluding those found in the controls. The volatiles regarded as insect attractants were then listed.

### Plant breeding system

Plant breeding system was studied using inter-simple sequence repeat (ISSR) markers. Leaf samples from three populations of each species were collected (*G. suaveolens*: 19 individuals from Rampayoh, 21 from Kuala Biang, and 20 from Beelaban; *G. tapisoides*: 20 individuals from Rampayoh, 16 from Teraja, and 19 from Andulau). DNA was extracted from c. 20 mg of homogenized silica-dried leaf tissue using DNeasy Plant Mini Kit (Qiagen, Hilden, Germany) following the manufacturer’s instructions, and stored at −20 °C. One hundred primers, 15–23 nucleotides in length (USB ISSR primers, oligonucleotide set 100/9; Biotechnology Laboratory, University of British Columbia, Vancouver) were screened. Eight primers (numbers 807, 808, 810, 822, 825, 834, 840 and 842) were selected for both species.

Single-primer PCR amplifications were conducted. A 25 μl mixture was prepared by mixing 0.3 μM primer, 2 units of Taq DNA polymerase (M8295, Promega, Madison, USA), 1× PCR buffer with 2.5 mM MgCl_2_, 0.2 mM dNTPs (R0192, Fermentas, Hong Kong) and 20 ng DNA. PCR amplifications were conducted using a GeneAmp PCR System 2700 (Applied Biosystems, California). The final products were stored at 4 °C before use.

PCR products were added to 1.8% agarose gels made with SYBR Safe DNA gel stain (Invitrogen, Hong Kong). PCR products were then transferred to the gel bathed in × 1 TAE buffer and run at 85 V for 65 min. A 100-bp DNA ladder (Fermentas, Hong Kong) was added for determining loci for each primer by comparing fragment sizes. The gels were then exposed under UV and bands recorded using a UVP gel documentation system. Bands were recorded as either present or absent, and the data matrix analysed using POPGENE ver. 1.32^35^ with the assumption that the populations are in Hardy-Weinberg equilibrium at all loci.

## Results

### Floral phenology

**Figure 4:**
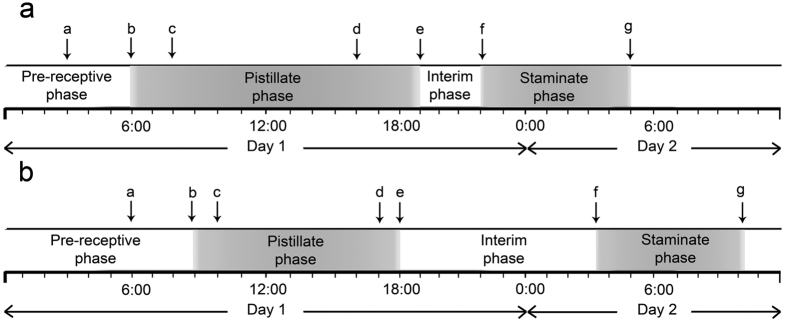
Phenological events in (**a**) *Goniothalamus tapisoides* and (**b**) *G. suaveolens*. a = rise of outer petals and opening of basal apertures; b = beginning of stigmatic exudate formation and scent emission; c = arrival of pollinators; d = closure of basal apertures; e = drying of exudate and lowering of scent emission; f = initiation of anther dehiscence; g = abscission of petals and departure of pollinators.

*Goniothalamus tapisoides* and *G. suaveolens* are both clearly protogynous, with anthesis of similar duration (23 h and 25 h, respectively), extending over two days (Fig. 4). Growth of the outer petals exposes the basal apertures between the inner petal ‘claws’, allowing pollinators to enter the otherwise enclosed floral chamber (*a* in Fig. 4). The pistillate phase begins in the morning with the formation of sticky stigmatic exudate (*b* in Fig. 4), associated with a sweet fruity scent, and continues until the early evening (*e* in Fig. 4). The apertures become blocked by the outer petals (trapping any potential pollinators) prior to the end of the pistillate phase (around dusk of the same day; *d* in Fig. 4). The apertures are closed as the anthers dehisce at the start of the staminate phase (*f* in Fig. 4) and remain closed until dawn the next day when the corolla abscises (*g* in Fig. 4). There is clear evidence of a non-sexual interim phase separating the pistillate and staminate phases of both species. There is no evidence of flowering synchrony in either species.

The floral phenological changes are described in detail below, highlighting the differences between the two species:

*Stage I: Pre-receptive phase.* The outer petals change from red-tinged to cream in *G. tapisoides* (Fig. 2a,b) and from green to yellow in *G. suaveolens* (Fig. 3a,b). The outer petals are initially compressed against the inner petal dome (Figs 2a and 3b), blocking the chamber apertures and hence preventing pollinators from accessing the floral reproductive organs. The outer petals subsequently begin to rise, exposing the apertures (Figs 2b and 3c) from 0300 hours in *G. tapisoides* and 0600 hours in *G. suaveolens (a* in Fig. 4).

**Figure 5:**
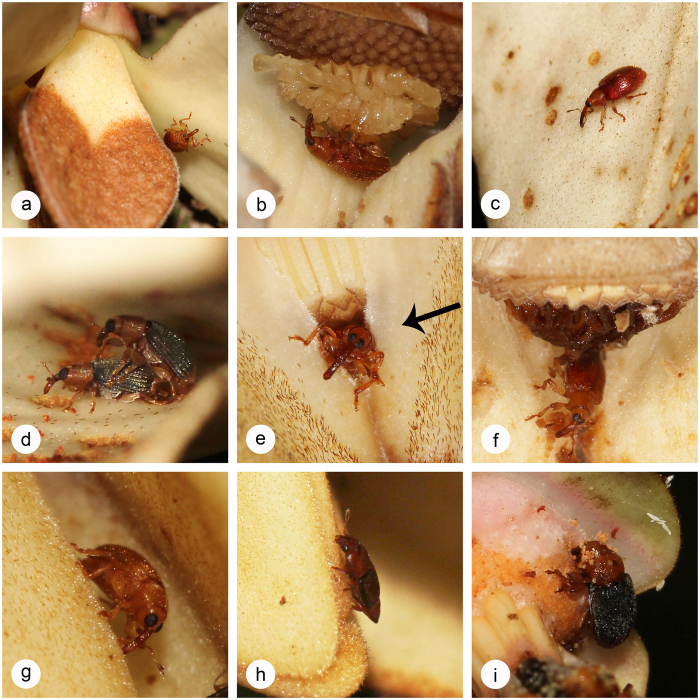
Activities of potential pollinators of *Goniothalamus tapisoides* (**a**–**f**) and *G. suaveolens* (**g–i**) during anthesis. (**a**) *Endaeus* sp. 1; (**b**) *Endaeus* sp. 1; (**c**) *Endaeus* sp. 2; (**d**) *Endaeus* sp. 3 copulating on the outer petals during pistillate phase; (**e**) the size of the basal apertures are near to the body size of an beetle individual(petals are removed) (**f**) beetle individual trapped during interim phase; (**g**) *Endaeus* sp. 1; (**h**) *Carpophilus* cf. *marginellus* Motschulsky; (**i**) cf. *Endaeus* sp.

*Stage II: Pistillate phase* (duration: 13 h in *G. tapisoides*; 9 h in *G. suaveolens*). The stigmas become receptive in the morning (0600 hours in *G. tapisoides*, 0900 hours in *G. suaveolens; b* in Fig. 4), as indicated by the formation of sticky stigmatic exudate (Figs 2e and 3e), which remains visible until that evening (1900 hours in *G. tapisoides*, 1800 hours in *G. suaveolens; e* in Fig. 4). The flowers emit distinctive floral odours (a sweet watermelon-like scent in *G. tapisoides*, and a strong banana-like scent in *G. suaveolens*) throughout this stage. The colour of the inner petals intensifies (from paler to darker brown) from 0900 hours in *G. tapisoides* (Fig. 2c), with the outer petals becoming brown after 1800 hours (Fig. 2f); no significant colour change was observed in *G. suaveolens* flowers, however. The apertures remain open during the early part of the pistillate phase (Figs 2c,d and 3d), allowing entry of pollinators (Fig. 5a,e). Subsequent growth of the outer petals closes these apertures from 1600 hours in *G. tapisoides* (Fig. 2f), and from 1700 hours in *G. suaveolens* (Fig. 3g; *d* in Fig. 4), effectively trapping pollinators inside the pollination chamber (Fig. 5f).

*Stage III: Interim phase* (duration: 3 h in *G. tapisoides*; 9 h in *G. suaveolens*). The stigmatic exudate begins to dry from 1900 hours in *G. tapisoides* and 1800 hours in *G. suaveolens (e* in Fig. 4). This drying is associated with browning of the carpels (Figs 2g,h, and 3f) and is indicative of the end of the pistillate phase: since the anthers have yet to dehisce it is clear that the flower is not sexually functional during the interim phase.

*Stage IV: Staminate phase* (duration: 7 h in both species). The onset of this phase is indicated by anther dehiscence (Figs 2i and 3h), which begins in the late evening (2200 hours) of the first day in *G. tapisoides* and in the early morning (0300 hours) of the second day in *G. suaveolens (f* in Fig. 4). The stamens partially abscise later, remaining suspended from the receptacle by extended tracheary elements (evident in Fig. 2j). The floral chamber remains tightly closed throughout, with the pollinators trapped inside. The staminate phase ends in the early morning (0500 hours) on the second day in *G. tapisoides* and later that morning (1000 hours) in *G. suaveolens*, following corolla abscission (Figs 2j and 3i; *g* in Fig. 4) when the pollinators are released.

### Pollinators and floral visitors

Pollinators of both species arrive in the morning (0800 hours in *G. tapisoides*, 1000 hours in *G. suaveolens; c* in Fig. 4). Thirteen different floral visitors belonging to five orders were recorded for *G. tapisoides*. Four of these species were small beetles that were retrieved from within the floral chamber (Table 1): three small curculionid beetles (*Endaeus* spp. 1–3: Supplementary Fig. S1a,b,d); and a small nitidulid beetle, tentatively identified as ‘cf. *Carpophilus*’ (Supplementary Fig. S1e). The other floral visitors were only observed outside the floral chamber, and included two rarely observed unidentified beetle species (both larger than the apertures in the floral chamber and therefore unable to access the floral reproductive organs; one individual observed per species), as well as ants (four species; a few individuals observed), and single observations each of fly, spider and cockroach species. Amongst these floral visitors, only the curculionid and nitidulid beetles are potential pollinators of *G. tapisoides* since they were retrieved from within the floral chamber and observed to pass through the apertures in the chamber (Fig. 5e).

The two most commonly observed beetle pollinators of *G. tapisoides* were *Endaeus* spp. 1 and 2 (Table 1). *Endaeus* sp. 1 was observed to feed on stigmatic exudate during the pistillate phase (Fig. 5b). Pollen grains were observed on the bodies of *Endaeus* sp. 2 (Supplementary Fig. S1c), retrieved from within the floral chamber of pistillate-phase flowers. *Endaeus* spp. 2 and 3 were observed copulating on the outer petals of pistillate-phase flowers (Fig. 5c,d). The ‘cf. *Carpophilus*’ beetle was observed to be active inside the floral chamber during the pistillate phase. The maximum number of beetle individuals found in a closed chamber was three.

Seven different floral visitors belonging to two orders were associated with *G. suaveolens*. Five of these species were beetles (Table 1), including three small curculionids (*Endaeus* spp. 1 and 2: Supplementary Fig. S1a,b; and a species tentatively classified as ‘cf. *Endaeus*’: Supplementary Fig. S1f), and two small nitidulids (*Carpophilus* cf. *marginellus* Motschulsky: Supplementary Fig. S1g; and *Carpophilus (Ecnomorphus*) cf. *dilutus* Motschulsky: Supplementary Fig. S1h). In addition to these five beetle species, which were all observed inside the floral chamber, two *Drosophila* species (Diptera) were observed to visit *G. suaveolens* flowers, although they did not enter the floral chamber. *Goniothalamus tapisoides* and *G. suaveolens* therefore share two potential pollinators: *Endaeus* spp. 1 and 2.

*Carpophilus* cf. *marginellus* (Fig. 5h) was the most abundant species observed inside *G. suaveolens* flowers, with up to 18 individuals in a single floral chamber; despite the frequency of these visits, there was no evidence of pollen grain attachment. The rarely encountered ‘cf. *Endaeus*’ species was observed to probe between stigmas and to bore holes into the receptacle after the end of the staminate phase and petal abscission (Fig. 5i). This species was notably seen carrying pollen grains (Fig. 5i). *Endaeus* sp. 1 was found near the apertures of pistillate-phase flowers (Fig. 5g) and also inside floral chambers.

### Floral thermogenesis and scent chemistry

Comparison of internal floral and ambient temperatures revealed no evidence of thermogenesis or heat retention in either species. *Goniothalamus tapisoides* flowers emit a weak watermelon-like scent during the pistillate phase, but become almost odourless during the interim and staminate phases. A total of 41volatile compounds were identified from the scents of mature (pistillate-, interim- and staminate-phase) and non-receptive flowers of *G. tapisoides* (Supplementary Table S1). Mature flowers of *G. suaveolens* emit a strong sweet banana scent, which is most intense during the pistillate phase. A total of 38 volatile compounds were recorded (Supplementary Table S2).

### Plant breeding system

A total of 150 ISSR loci were recorded in *G. tapisoides*, with bands 250–2072 bp in length, and with 3–22 bands (averaging 11.4) per primer. Within-population gene diversity (*H*_*S*_) was 0.17 ± 0.02, whilst total genetic diversity in pooled populations (*H*_*T*_) was 0.19 ± 0.02 (Table 2): the three populations are therefore genetically very similar, since within-population gene diversity contributes c. 90% of total genetic diversity. The genetic similarity of populations is likely to have resulted from considerable gene flow between populations (*N*_*m*_ = 3.67). This is also reflected by the high average gene identity (*I* = 0.96) and the low average genetic distance between population (*D* = 0.042) despite the considerable geographical distance separating populations: Andulau and Jalan Rampayoh are 30.9 km apart, and Andulau and Teraja are 36.0 km apart. The coefficient of genetic differentiation between populations (*G*_*ST*_), an indicator of breeding system, is 0.12 in *G. tapisoides*. The percentage of polymorphic loci (*P*), mean observed number of alleles (*A*), effective number of alleles (*A*_*e*_) and gene diversity (*h*) of *G. tapisoides* are 67%, 1.67, 1.27 and 0.17, respectively.

Corresponding data for *G. suaveolens* revealed 150 ISSR loci, with band sizes ranging from 250–2072 bp and 7–20 bands (averaging 12.5) per primer. Within-population gene diversity (*H*_*S*_) and total genetic diversity in pooled populations (*H*_*T*_) are 0.17 ± 0.01 and 0.23 ± 0.02, respectively (Table 2), with the former representing c. 75% of total genetic diversity. The average gene identity (*I*) was 0.89 whilst the average genetic distance (*D*) was 0.12, with considerable geographical separation between populations: Rampayoh and Kuala Biang are 77.5 km apart, whereas Rampayoh and Beelaban are 63.0 km apart. The Level of gene flow between populations (*N*_*m*_) was 1.35, and the coefficient of genetic differentiation between populations (*G*_*ST*_) was 0.27. The percentage of polymorphic loci (*P*), mean observed number of alleles (*A*), effective number of alleles (*A*_*e*_) and gene diversity (*h*) of *G. suaveolens* are 61.3%, 1.61, 1.28 and 0.17, respectively.

## Discussion

### Floral biology, pollination ecology and breeding system

*Goniothalamus tapisoides* flowers are protogynous with 23-h anthesis, beginning in the early morning (0600 hours) and ending the following morning (0500 hours). As with all hermaphroditic-flowered Annonaceae species^36^, the flowers are protogynous. There is evidence of a 3-h non-sexual interim phase separating the pistillate and staminate phases, which would effectively preclude autogamy. Although pistillate/staminate-phase floral synchrony (in which flowers on an individual are synchronised in order to avoid maturation on consecutive days, hence avoiding geitonogamy) is likely to be widespread in Annonaceae^37^, there is no evidence for such synchrony in *G. tapisoides*: 25% of flowers are borne on the same tree on consecutive days. The likelihood of geitonogamy occurring in this species is minimised, however, by the limited number of mature flowers borne on the same individual on consecutive days, and it seems probable that *G. tapisoides* is predominantly xenogamous. This conclusion is supported by the results of the ISSR analysis: within-population gene diversity (*H*_*S*_) contributes c. 90% of total genetic diversity (Table 2), possibly due to the high level of gene flow between populations (*N*_*m*_ = 3.67). The coefficient of genetic differentiation between populations (*G*_*ST*_ = 0.12), indicates probable xenogamy. The average gene identity (*I*) and average genetic distance (*D*) furthermore suggest that the three populations are genetically similar, possibly due to the integrity of forests in Brunei, with 54% of land area covered by unlogged forests^38^, facilitating pollinator and frugivore movement.

A small curculionid beetle (*Endaeus* sp. 2) is inferred as the effective pollinator of *G. tapisoides*, evidenced by: (1) the presence of pollen grains on the beetles; (2) the abundance of this species on and around mature flowers (observations in 2014); and (3) evidence of inter-floral movement, demonstrated by the presence of pollen-laden beetles in pistillate-phase flowers. Although *Endaeus* sp. 2 is implicated as an effective pollinator, *Endaeus* sp. 1 is also a potential pollinator since numerous floral visits were recorded in 2013. Annonaceae species rarely evolve species-specific pollination systems^21^: in most cases the specialisation is only restricted to pollinator guilds, with different representatives of the guild acting as primary pollinator in different flowering seasons and in different populations.

*Endaeus* beetles are likely to be attracted to *G. tapisoides* flowers by the watermelon-like scent that is emitted during the pistillate phase. GC-MS analysis of the scent revealed four volatiles that have been implicated as chemical signals (attractants, allomones, kairomones or pheromones) for Coleoptera. Three compounds (1,3,3-Trimethyl-2-oxabicyclo[2.2.2]octane, phenylmethanol and phenol) were found to be semiochemicals for Curculionidae^39^,^40^,^41^,^42^,^43^ (Table 3), which are the main pollinators of *G. tapisoides* (although it should be noted that 1,3,3-trimethyl-2-oxabicyclo[2.2.2]octane was only recorded from staminate-phase flowers, and is therefore unlikely to function as an attractant in *G. tapisoides*). The attractant phenylmethanol, which is only produced during the pistillate phase, has a sweet aroma, previously recorded in papaya fruits^44^ and is therefore likely to contribute to the fruity component of the floral scent. The scent dissipates towards the end of the pistillate phase, but by that stage the apertures in the floral chamber are obstructed by the movement of the outer petals, effectively trapping the beetles within the chamber (discussed below). The beetles are initially likely to be rewarded by stigmatic exudate: *Endaeus* sp. 2 was observed to feed on exudate, supporting previous reports for *Endaeus* on flowers of other Annonaceae species^26^. This food source would become unavailable to beetles during the staminate phase, but again it is noteworthy that the beetles are trapped within the floral chamber at this stage.

*Goniothalamus suaveolens* flowers are similarly protogynous with 25-h anthesis, from 0900 hours until 1000 hours the following morning. As with *G. tapisoides*, the pistillate and staminate phases in *G. suaveolens* flowers are separated by a non-sexual interim phase; this phase is considerably longer than that of *G. tapisoides*, extending over 9 h, but similarly functions to preclude autogamy. Pistillate/staminate-phase floral synchrony was not observed (17% of flowers were borne on the same tree on consecutive days), and hence geitonogamy is possible. As with *G. tapisoides*, however, the likelihood of geitonogamy is reduced by the limited number of mature flowers borne on the same individual on consecutive days. The conclusion that *G. suaveolens* is predominantly xenogamous is also be supported by the ISSR data: within-population gene diversity (*H*_*S*_) contributes c. 75% of the total diversity (Table 2). Although the coefficient of genetic differentiation between populations (*G*_*ST*_) in *G. suaveolens* was 0.27 (typical for mixed-mating species), the level of gene flow between populations (*N*_*m*_ = 1.35) was high: average *N*_*m*_ values for mixed-mating and outcrossing species are 0.727 and 1.154, respectively^45^. Evidence for outcrossing is also provided by the gene identity (*I*) and genetic distance (*D*) values which suggest that the three populations are genetically similar despite the considerable distance between populations and that they may have originated from a common ancestral population.

All five species of beetle retrieved from within the floral chamber of *G. suaveolens* are potential pollinators. Although two of these species are shared with *G. tapisoides (Endaeus* spp. 1 and 2), these species are more likely to be the effective pollinators of *G. tapisoides* than *G. suaveolens* because of their very infrequent visits to the latter. *Carpophilus* cf. *marginellus* (Nitidulidae), however, was likely to be an effective pollinator of *G. suaveolens* given its frequent visits to mature flowers. Six volatiles identified from the floral scent of *G. suaveolens* are reported to be semiochemicals for Coleoptera, with four regarded as specific attractants to Nitidulidae or Curculionidae (Table 3). Among these, 3-methylbutyl acetate, ethyl hexanoate and 2-phenylethanol, which are reported as attractants or kairomones to Nitidulidae^46^,^47^,^48^ and were only released by *G. suaveolens* flowers during the pistillate phase, are noticeably absent from the floral scent of *G. tapisoides*: this may explain the infrequency of nitidulid visits to *G. tapisoides* flowers. 2-phenylethanol has been reported as a kairomone for *Carpophilus*^48^, which is the genus identified as the major pollinator for *G. suaveolens*. Several curculionid attractants are present in the floral scents of both *Goniothalamus* species, possibly explaining visits by *Endaeus* spp. 1 and 2 to both. It is also noteworthy that 3-methylbutyl acetate and ethyl hexanoate are aliphatic esters associated with fruity odours^49^,^50^,^51^: the former, which is a key component of banana scent^50^, was only recorded during the pistillate phase, when the banana-like scent was evident.

### Pollinator trapping

The pollination chamber in *Goniothalamus* flowers is formed by apically connivent and postgenitally fused inner petals, with small apertures located between the basal ‘claws’ of the inner petals that are sometimes blocked by the outer petals that alternate with the inner petals. This type of chamber (‘type III’^52^) is relatively common in Annonaceae, and is hypothesised to have evolved independently in several lineages^52^. Floral chambers are believed to serve several functions in the family, including maintenance of a micro-environment optimized for pollinators (e.g., retaining floral heat) and providing a protected feeding, copulation and/or ovipositing site. Although essentially closed, the floral chamber generally does not prevent the arrival or departure of floral visitors^37^.

We present evidence of a ‘trapping’ mechanism that effectively prevents beetles from leaving the flower once the floral chamber has closed: this is the first time that such a mechanism has been reported in Annonaceae. Pollinator trapping in *G. tapisoides* and *G. suaveolens* occurs before the end of the pistillate phase (*d* in Fig. 4): the beetle pollinators therefore cannot leave the flower until petal abscission (*g* in Fig. 4), after the end of the staminate phase. Trapping in *Goniothalamus* is achieved by growth of the outer petals so that the adaxial surface of each petal is compressed against the apertures in the inner petal dome: the margins of the inner petal claws are glabrous (Fig. 5e) and are therefore able to closely compress against the corresponding glabrous region on the adaxial surface of the outer petals (Fig. 2k). The margins of the inner petal claws are often extended to form a thin extended flange in *Goniothalamus tapisoide*s (Fig. 2l; see also *G. tomentosus*^53^), enhancing the intimacy of the connection. Field observations of *G. tapisoides* and *G. suaveolens* suggest that the trapping mechanism is particularly effective in the former due to the presence of an extended flange and the additional growth of the outer petals, further blocking the apertures. It is noticeable that the pollination chamber in *G. tapisoides* cannot easily be opened by artificially raising the outer petals; the trap in *G. suaveolens* flowers, in contrast, is looser. It seems unlikely that trapped beetles would be able to leave closed chambers in either species, and this was never observed during our field observations. Based on morphological assessment of other species in the genus, it appears that this trapping mechanism is common to all species and is likely to be a key evolutionary innovation of considerable functional significance.

Anthesis in Annonaceae normally extends over three days (36–50 h): e.g., 36 h in *Huberantha korinti* (as ‘*Polyalthia korinti*’)^22^ and *Monoon coffeoides* (as ‘*Polyalthia coffeoides*’)^22^; 39 h in *Mitrephora heyeana*^54^ and *Xylopia championii*^55^; 36–50 h in *Annona coriacea*^56^; 37–43 h in *Uvariodendron connivens*^57^; 38–39 h in *Uvariodendron calophyllum*^57^; 50 h in *Uvaria elmeri*^58^; 30–54 h in *Piptostigma* species^57^; and c. 3 days in *Monodora tenuifolia*^57^. In these cases, the timing and duration of the pistillate phase on the first day of anthesis is similar to that of the generally staminate phase the next day, and as a result pistillate- and staminate-phase flowers can co-exist without significantly encouraging geitonogamy: pollen-laden beetles departing from post-staminate-phase flowers (in the late evening) would not be attracted to other flowers entering the pistillate phase until early the following morning, and this time difference would enhance opportunities for pollinator movement between different trees, albeit subject to attrition costs associated with pollinator loss.

The two *Goniothalamus* species studied here have compressed anthesis (23 h in *G. tapisoides*; 25 h in *G. suaveolens*); similarly abbreviated anthesis has been recorded in *Dasymaschalon* (26 h)^37^. Anthesis commonly extends over two days in early-divergent angiosperms, with the pistillate phase usually starting in the morning and the staminate phase ending in the morning of the next day^59^. Abbreviated anthesis may provide a selective advantage by increasing the turnover rate of flowers and hence promoting fruitset. One of the consequences of compressed anthesis, however, is that the different sexual phases of the flowers will occur at different times of the day (Fig. 4). Interfloral movement of beetles between staminate- and pistillate-phase flowers is optimized, however, by delaying beetle departure from flowers until the onset of the pistillate phase in other flowers. Pollinator trapping in species with short anthesis not only enhances pollination efficiency by increasing the likelihood of interfloral pollen transfer, but also increases the time during which the pollinators inside staminate-phase flowers can inadvertently collect pollen. Since the pollinators become trapped within flowers prior to the end of the pistillate phase, there is no selective advantage for the plant in continuing to provide pollinator attractants or rewards: the plant is therefore able to limit its reproductive investment by terminating floral scent production much earlier than is typical in the family. Pollinator trapping is widespread in the genus, including *G. malayanus* Hook. f. & Thomson, *G. parallelivenius* Ridl. and *G. velutinus* (pers. observ.).

### Coevolution and partitioning of pollination resources

This is the first study focusing on the evolution of reproductive isolation between sympatric species of early-divergent angiosperm. We provide evidence that supports the hypothesis that pollinator resources are partitioned among two sympatric congeners, *Goniothalamus suaveolens* and *G. tapisoides*. Partitioning is mainly achieved by ethological and temporal isolation, both of which are likely to be effective in reducing interspecific pollen transfer.

Ethological isolation between these species can be inferred from the chemical analysis of floral scents. The flowers of these species emitted distinctive scents: watermelon-like in the case of *G. tapisoides*, and banana-like in *G. suaveolens*. Although these scents were shown to include many volatiles that are generalist coleopteran attractants, the GC-MS analysis also provides evidence of specific specialisation for curculionid and nitidulid beetles (Table 3), which are both frequent floral visitors to the two species (Table 1). *Goniothalamus suaveolens* flowers, which are primarily pollinated by *Carpophilus* beetles (Nitidulidae), contains three volatiles that have been identified as kairomone or attractants for nitidulids (Table 3), viz. 3-methylbutyl acetate^60^, ethyl hexanoate^47^ and 2-phenylethanol^47^,^48^. In marked contrast, the floral scent of *G. tapisoides* did not contain volatiles that have previously been linked specifically with nitidulids (Table 3). The effective pollinators of *G. tapisoides* are likely to be *Endaeus* beetles (Curculionidae), and chemical analysis of the floral scent revealed three volatiles (1,3,3-trimethyl-2-oxabicyclo[2.2.2]octane, phenylmethanol, and phenol) that have previously been identified as semiochemicals for curculionids^39^,^40^,^41^,^42^,^43^ (Table 3).

Flower-level phenology also contributes to reproductive resource partitioning among these species. *Goniothalamus tapisoides* flowers are sexually functional for 23 h from 0600 hours, whereas *G. suaveolens* flowers are functional for 25 h from 0900 hours. The floral phenology of the two species are separated by several hours: pollen-laden beetles leaving *G. tapisoides* flowers at the end of the staminate phase around 0500 hours (*g* in Fig. 4a) would be attracted to pistillate-phase flowers that become functional shortly after (from 0600 hours; b in Fig. 4a); in *G. suaveolens*, however, the pollen-laden beetles leaving post-staminate flowers around 1000 hours (*g* in Fig. 4b) would be attracted to pistillate-phase flowers that are already functional (0900 hours; *b* in Fig. 4b). Since the beetles presumably respond immediately to other attractants, it is likely that beetles would move between conspecific flowers rather than moving between different species.

These two sympatric and phylogenetically closely related *Goniothalamus* species therefore show reproductive resource partitioning as a result of within-flower phenological differences (in which anthesis is separated by a few hours) and the production of floral scents that are specific to different beetle families. Another key observation is that these two *Goniothalamus* species possess abbreviated anthesis of 23–25 h, correlated with a previously undescribed trapping mechanism. This mechanism strengthens pollination efficiency by increasing the likelihood of interfloral pollen transfer and the time that beetles are able to collect pollen during the staminate phase.

## Additional Information

**How to cite this article**: Lau, J. Y. Y. *et al*. Reproductive resource partitioning in two sympatric *Goniothalamus* species (Annonaceae) from Borneo: floral biology, pollinator trapping and plant breeding system. *Sci. Rep.* **6**, 35674; doi: 10.1038/srep35674 (2016).

## Supplementary Material

Supplementary Information

## Figures and Tables

**Figure 1:**
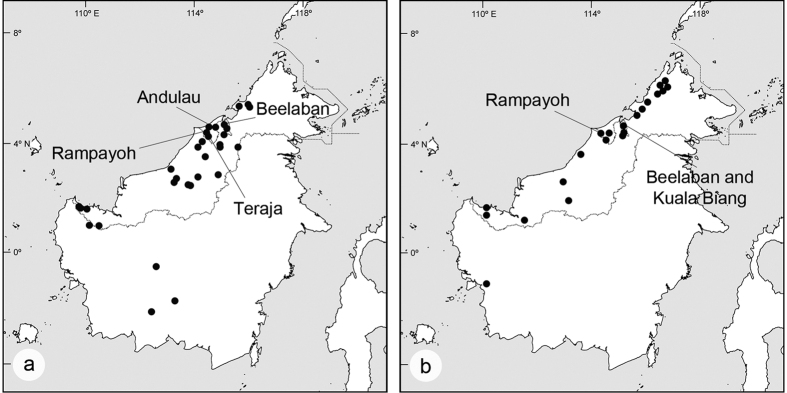
Distributions of (**a**) *Goniothalamus tapisoides* and (**b**) *G. suaveolens* in Borneo and the location of field sites.

**Table 1:** Number of beetle visitors found in *G. tapisoides* and *G. suaveolens* in 2013 and 2014.

Plant species	Floral visitor	2013	2014
*G. tapisoides*		(*n* = 39)	(*n* = 161)
	Curculionidae		
	*Endaeus* sp. 1	33	20
	*Endaeus* sp. 2	0	22
	*Endaeus* sp. 3	3	13
	Nitidulidae		
	cf. *Carpophilus* sp.	6	11
*G. suaveolens*		(*n* = 24)	(*n* = 6)
	Curculionidae		
	*Endaeus* sp. 1	3	1
	*Endaeus* sp. 2	4	0
	cf. *Endaeus* sp.	2	0
	Nitidulidae		
	*Carpophilus* cf. *marginellus*	14	18*
	*Carpophilus* cf. *dilutus*	1	0

*n* = number of flowers examined.^*^Multiple beetles observed from a single flower.

**Table 2:** Genetic parameters within and among populations in *Goniothalamus tapisoides* and *G. suaveolens*.

Genetic parameters	*Goniothalamus tapisoides*				*Goniothalamus suaveolens*			
	Rampayoh	Teraja	Andulau	Pooled populations	Rampayoh	Kuala Biang	Beelaban	Pooled populations
*n*	20	16	19	54	19	21	20	59
*P* (%)	58	66	77	100	58	71	55	99
*A* ± S. D.	1.58 ± 0.50	1.66 ± 0.47	1.77 ± 0.42	2.00 ± 0.00	1.58 ± 0.50	1.71 ± 0.46	1.55 ± 0.50	1.99 ± 0.12
*A*_*e*_* ± S. D.*	1.23 ± 0.31	1.25 ± 0.28	1.32 ± 0.34	1.29 ± 0.29	1.25 ± 0.34	1.30 ± 0.33	1.29 ± 0.36	1.36 ± 0.30
*H*_*o*_* ± S. D.*	0.23 ± 0.25	0.27 ± 0.23	0.31 ± 0.25	0.32 ± 0.21	0.23 ± 0.26	0.29 ± 0.25	0.26 ± 0.28	0.37 ± 0.21
*h ± S. D.*	0.14 ± 0.17	0.17 ± 0.16	0.20 ± 0.18	0.19 ± 0.15	0.15 ± 0.18	0.18 ± 0.18	0.17 ± 0.19	0.23 ± 0.16
*H*_*S *_*± S. D.*	—	—	—	0.17 ± 0.02	—	—	—	0.17 ± 0.01
*H*_*T*_* ± S. D.*	—	—	—	0.19 ± 0.02	—	—	—	0.23 ± 0.02
*D*_*ST*_	—	—	—	0.02	—	—	—	0.062
*G*_*ST*_	—	—	—	0.12	—	—	—	0.27
*N*_*m*_	—	—	—	3.67	—	—	—	1.35
*I*	—	—	—	0.96	—	—	—	0.89
*D*	—	—	—	0.042	—	—	—	0.12

*n* = sample size; *P* = percentage of polymorphic loci; *A* = mean observed number of alleles; *A*_e_ = effective number of alleles; *H*_*o*_ = Shannon’s information index; *h* = gene diversity; *H*_*S*_ = within-population gene diversity; *H*_*T*_ = total genetic diversity in pooled populations; *D*_*ST*_ = total genetic diversity distributed among populations; *G*_*ST*_ = coefficient of genetic differentiation between populations = (*H*_*T*_ − *H*_*S*_)/*H*_*T*_; *N*_*m*_ = level of gene flow between populations; *I* = average gene identity and *D* = average genetic distance.

**Table 3:** Putative chemicals recorded in flowers of *Goniothalamus tapisoides* and *G. suaveolens* that serve as chemical signals to different groups of floral visitors (according to Pherobase^61^).

Species & compound names	Mean RT (min)	Curculionidae	Nitidulidae	Other Coleoptera^61^	Phenological phase
***Goniothalamus tapisoides***					
Bicyclic monoterpenoid ethers					
1,3,3-Trimethyl-2-oxabicyclo[2.2.2]octane	8.11	A^39^, Al^43^		A, Al	S
Benzenoid alcohols					
Phenylmethanol	25.94	A^42^, K^40^		A, Al, K	P
Benzenoids					
Phenol	28.54	K^41^		A, Al, P	N, P, I, S
Fatty acids					
Hexadecanoic acid	32.58			A, Al, K, P	I, S
***Goniothalamus suaveolens***					
Aliphatic esters					
3-methylbutyl acetate	5.19	P^60^	A^46^,^47^	A, K	P
Ethyl hexanoate	9.15		A^47^		P
Aliphatic hydrocarbons					
Hexadecane	19.70			Al, P	I,S
Benzenoid alcohols					
2-phenylethanol	26.66	A^42^, K^41^	A^47^, K^48^	A, Al, K, P	P
Benzenoids					
Phenol	28.55	K^41^		A, Al, P	P, S
Fatty acids					
Hexadecanoic acid	32.90			A, Al, K, P	P, I, S

Abbreviations: A = attractant; Al = allomone; K = kairomone; P = pheromone. The compounds are corresponding to those in Supplementary Tables S1 and S2. Chemical compounds that belong to the same chemical class are arranged in ascending retention time (RT). Phenological phases: N = non-receptive phase; P = pistillate phase; I = interim phase; S = staminate phase.
