# Supplementary material for: Reproductive resource partitioning in two sympatric Goniothalamus species (Annonaceae) from Borneo: floral biology, pollinator trapping and plant breeding system
Source: Sci Rep. 2016 Oct 21;6:35674. doi: 10.1038/srep35674 (PMC5073367; doi:10.1038/srep35674)

Reproductive resource partitioning in two sympatric *Goniothalamus* species from Borneo: floral biology, pollinator trapping and plant breeding system

Jenny Y. Y. Lau, Chun Chiu Pang, Lawrence Ramsden, Richard M.K. Saunders*

School of Biological Sciences, The University of Hong Kong, Pokfulam Road, Hong Kong, China. Correspondence and requests for materials should be addressed to R.M.K.S. (email: saunders@hku.hk)

Supplementary Table S1. Putative chemical composition of floral volatile emitted by flowers of *Goniothalamus tapisoides* following exclusion of chemicals retrieved from blank controls. Phenological phases: N = non-receptive phase; P = pistillate phase; I = interim phase; S = staminate phase. Compounds arranged according to retention time (RT).

No.	Compound name	Mean RT (min)	Identity likelihood				Percentage of total peak area			
			N	P	I	S	N	P	I	S
1	4-isopropenyl-1-methylcyclohexene	7.71	86	...	...	...	0.07	...	...	...
2	(4R)-1-methyl-4-prop-1-en-2-ylcyclohexene	7.73	...	94	...	...	...	0.05	...	...
3	1,3,3-Trimethyl-2-oxabicyclo[2.2.2]octane	8.11	...	...	...	98	...	...	...	0.38
4	Docosane	9.17	...	...	...	80	...	...	...	0.2
5	2,6,11-trimethyldodecane	10.02	90	90	86	<80	2.82	0.26	1.13	1.93
6	1-Isopropyl-2-methylbenzene	10.18	...	97	...	...	...	0.14	...	...
7	4-Isopropylidene-1-methylcyclohexene	10.62	...	94	...	...	...	0.05	...	...
8	5-ethyl-2-methyloctane	11.37	...	...	...	81	...	...	...	1.85

9	4,6,8-Trimethyl-1-nonene	11.98	...	86	...	...	...	0.38	...	...
10	2,6,10-trimethyldodecane	12.17	...	...	83	...	...	...	0.24	...
11	2,6,10,15-tetramethylheptadecane	13.71	91	...	...	...	0.15	...	...	...
12	1,4-dichlorobenzene	15.38	97	98	90	96	0.14	0.09	0.08	0.23
13	7β-H-Silfiperfol-5-ene	15.59	...	90	...	...	...	0.07	...	...
14	2,6,11,15-tetramethylhexadecane	17.32	...	...	...	86	...	...	...	0.25
15	3,7-dimethylocta-1,6-dien-3-ol	18.46	...	...	...	96	...	...	...	0.59
16	4,6-dimethyl-6-(4-methylpent-3-enyl)bicyclo[3.1.1]hept-3-ene	18.81	...	...	...	98	...	...	...	0.47
17	6,7-dimethyl-7-(4-methylpent-3-enyl)-2,3,4,5-tetrahydro-1H-tricyclo[2.2.1.0^{2,6}]heptane	18.84	...	...	98	...	...	...	0.49	...
18	(1S,5S,6S)-2,6-Dimethyl-6-(4-methyl-3-penten-1-yl)bicyclo[3.1.1]hept-2-ene	19.21	...	...	97	99	...	...	1.33	1.39
19	4-methyl-1-propan-2-ylcyclohex-3-en-1-ol	19.72	...	...	...	95	...	...	...	0.39
20	10,10-Dimethyl-2,6-dimethylenbicyclo[7.2.0]undecane	19.89	...	98	...	...	...	0.41	...	...
21	2-(2-ethoxyethoxy)ethanol	20.22	...	...	86	80	...	...	0.2	0.16
22	(4R,4aS,6R)-6-Isopropenyl-4,4a-dimethyl-1,2,3,4,4a,5,6,7-octahydronaphthalene	20.74	...	83	...	...	...	0.59	...	...
23	2-Isopropenyl-4a,8-dimethyl-1,2,3,4,4a,5,6,7-octahydronaphthalene	21.37	97	...	...	...	0.76	...	...	...
24	5,8a-dimethyl-3-propan-2-ylidene-1,2,4,4a,7,8-hexahydronaphthalene	22.12	93	91	...	...	12.64	23.92	...	...
25	(3S,8aS)-5,8a-dimethyl-3-prop-1-en-2-yl-2,3,4,4a,7,8-hexahydro-1H-naphthalene	22.48	96	...	...	...	0.73	...	...	...
26	Naphthalene	22.76	95	...	...	...	9.26	...	...	...
27	(8aS)-8a-methyl-5-methylidene-3-prop-1-en-2-yl-1,2,3,4,4a,8-hexahydronaphthalene	23.01	...	91	...	...	...	7.87	...	...
28	Methyl (Z)-N-hydroxybenzenecarboximidate	23.68	87	90	<80	90	1.09	0.47	0.41	1.22

29	5,8a-dimethyl-3-prop-1-en-2-yl-2,3,7,8-tetrahydro-1H-naphthalene	24.23	95	99	...	...	0.43	3.55	...	...
30	Propan-2-yl dodecanoate	25.06	99	...	...	<80	0.15	...	...	0.17
31	Bicyclo[4.4.1]undeca-1,3,5,7,9-pentaene	25.19	93	...	...	...	0.07	...	...	...
32	Phenylmethanol	25.94	...	98	...	...	...	0.53	...	...
33	1,3-dimethylnaphthalene	28.24	91	...	...	...	0.05	...	...	...
34	1,7-dimethylnaphthalene	28.25	...	...	...	94	...	...	...	0.05
35	Phenol	28.54	94	93	90	94	0.37	0.18	0.18	0.34
36	2-phenoxyethanol	31.18	93	...	...	87	0.13	...	...	0.13
37	Ethyl 4-ethoxybenzoate	31.63	98	...	94	98	...	0.25	0.18	0.31
38	Hexadecanoic acid	32.58	...	...	<80	94	...	...	0.1	0.11
39	Dimethyl benzene-1,2-dicarboxylate	33.95	93	...	...	91	0.13	...	...	0.09
40	2,4-ditert-butylphenol	34.35	...	95	...	...	...	2.07	...	...
41	1,4a-dimethyl-7-prop-1-en-2-yl-3,4,5,6,7,8-hexahydronaphthalen-2-one	34.8	99	99	...	...	0.68	0.82	...	...

Supplementary Table S2. Putative chemical composition of floral volatile emitted by flowers of *Goniothalamus suaveolens* following exclusion of chemicals retrieved from blank controls. Phenological phases: N = non-receptive phase; P = pistillate phase; I = interim phase; S = staminate phase. Compounds arranged according to retention time (RT).

No.	Compound name	Mean RT (min)	Identity likelihood				Percentage of total peak area			
			N	P	I	S	N	P	I	S
1	3-methylbutyl acetate	5.19	...	90	...	...	...	1.65	...	...
2	4-methyl-1-propan-2-ylbicyclo[3.1.0]hex-2-ene	6.57	...	...	80	...	...	...	0.11	...
3	Nonane	8.85	...	83	...	...	...	0.06	...	...
4	Ethyl hexanoate	9.15	...	93	...	...	...	0.14	...	...
5	2,6,11-trimethyldodecane	9.84	...	81	83	<80	...	0.62	2.3	0.33
6	1,4-dichlorobenzene	15.41	...	94	96	...	...	0.06	0.07	...
7	2-bromododecane	16.63	...	90	...	...	...	0.28	...	...
8	(1S,5S,6R)-4,6-dimethyl-6-(4-methylpent-3-enyl)bicyclo[3.1.1]hept-3-ene	19.22	...	93	...	...	...	0.08	...	...
9	Hexadecane	19.7	...	...	96	86	...	...	0.46	0.16
10	2-(2-ethoxyethoxy)ethanol	20.23	...	...	<80	90	...	...	0.33	0.2
11	2,6,10,15-Tetramethylheptadecane	21.24	...	80	<80	...	...	0.12	0.32	...
12	2,6,10-trimethyltetradecane	21.32	...	...	90	...	...	...	0.32	...
13	[(2E)-3,7-dimethylocta-2,6-dienyl] acetate	23.35	...	91	...	...	...	0.58	...	...
14	Methyl (Z)-N-hydroxybenzenecarboximidate	23.68	...	87	...	<80	...	0.89	...	0.52
15	2-phenylethyl acetate	24.58	...	90	...	...	...	0.3	...	...
16	2,6,10,14-Tetramethylhexadecane	24.96	...	...	83	...	...	...	0.26	...
17	Bicyclo[4.4.1]undeca-1,3,5,7,9-pentaene	25.06	...	97	...	...	...	0.17	...	...

18	Propan-2-yl dodecanoate	25.09	...	...	99	87	...	...	3.41	0.23
19	1-methylnaphthalene	25.19	...	94	...	...	...	0.09	...	...
20	(2E)-3,7-dimethylocta-2,6-dien-1-ol	25.4	...	96	...	...	...	0.28	...	...
21	2-phenylethanol	26.66	...	93	...	...	...	0.18	...	...
22	1,7-dimethylnaphthalene	28.27	...	...	97	...	...	...	0.18	...
23	Phenol	28.55	...	94	...	91	...	0.33	...	0.25
24	Tetratetracontane	29.97	95	...	...	...	0.53	...	...	...
25	2-nitroethylbenzene	30.63	...	...	...	96	...	...	...	1.55
26	2-phenoxyethanol	31.19	...	87	...	87	...	0.33	...	0.09
27	Ethyl 4-ethoxybenzoate	31.64	...	93	98	96	...	0.4	1.54	0.3
28	Hexadecanoic acid	32.9	...	96	97	94	...	0.28	0.17	0.08
29	Hexadecan-1-ol	33.02	95	...	...	...	1.64	...	...	...
30	Dimethyl benzene-1,2-dicarboxylate	33.97	...	90	94	95	...	0.16	0.13	0.22
31	Methyl octyl phthalate	35.07	90	...	...	...	0.46	...	...	...
32	Octadec-1-ene	35.22	96	...	...	...	0.5	...	...	...
33	1-hexacosene	36.01	93	...	...	...	0.43	...	...	...
34	Diphenylmethanone	37.01	...	...	87	...	...	...	0.11	...
35	3,5-ditert-butyl-4-hydroxybenzaldehyde	37.27	...	...	94	...	...	...	0.14	...
36	1-O-butyl 2-O-tetradecyl benzene-1,2-dicarboxylate	38.08	...	...	81	...	...	...	0.42	...
37	Octadecan-1-ol	38.32	95	...	...	...	2.48	...		...
38	2-octyldodecan-1-ol	39.51	98	...	...	...	0.81	...		...

Supplementary Fig. S1 Pollinators of *Goniothalamus tapisodies* and *G. suaveolens*. a, *Endaeus* sp. 1; b, *Endaeus* sp. 2; c, pollen grains of *G. tapisoides* deposited on *Endaeus* sp. 2; d, *Endaeus* sp. 3; e, cf. *Carpophilus* sp.; f, cf. *Endaeus* sp. ; g, *Carpophilus* cf. *marginellus* Motschulsky; h, *Carpophilus* (*Ecnomorphus*) cf. *dilutus* Motschulsky. (Background artificially darkened using Photoshop graphics software).

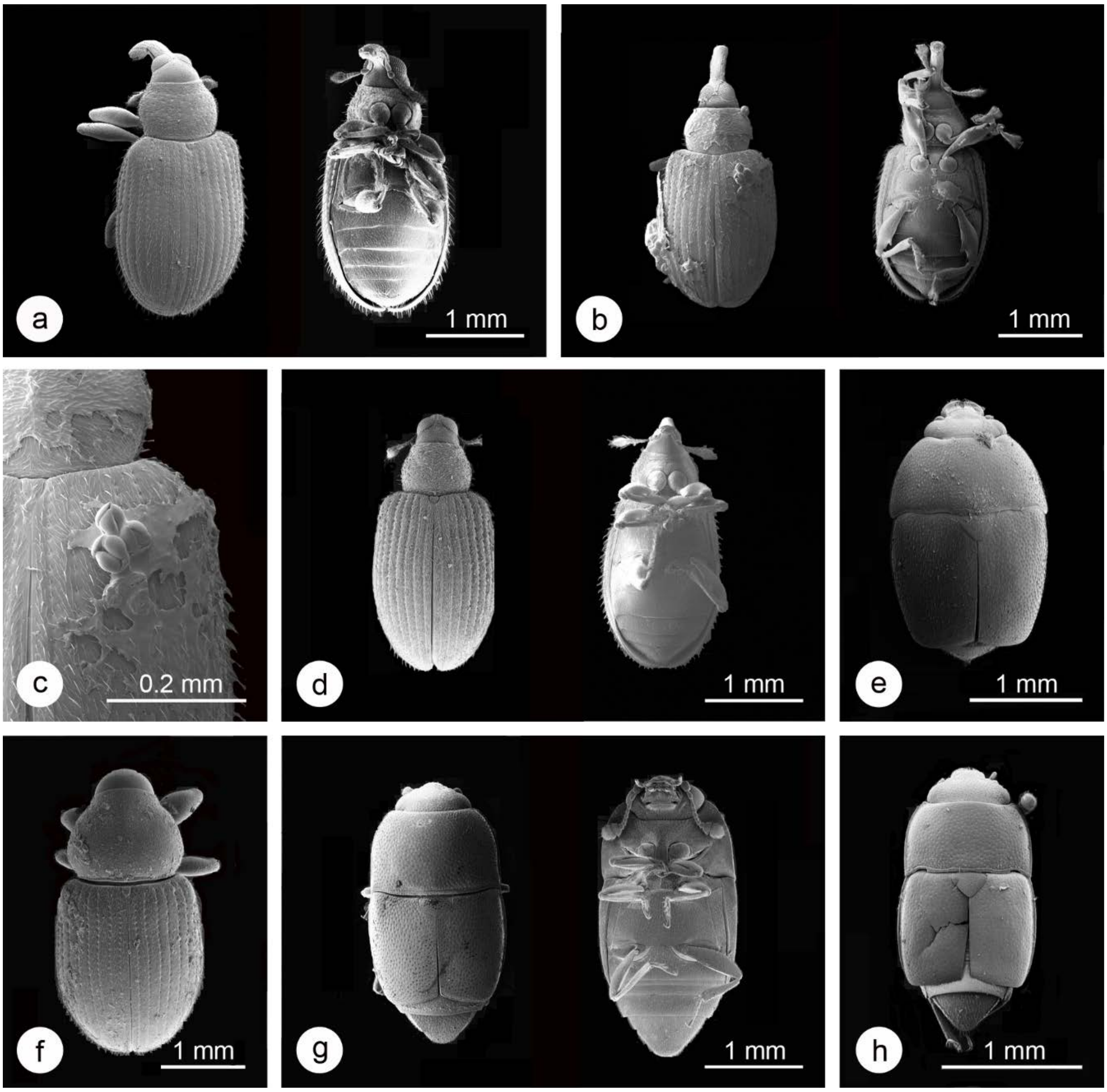

Supplement: Supplementary Information [file srep35674-s1.pdf]
